# Relationship between serum apolipoprotein B and risk of all-cause and cardiovascular disease mortality in individuals with hypertension: a prospective cohort study

**DOI:** 10.1186/s12872-024-03949-1

**Published:** 2024-05-24

**Authors:** Ying Huang, Siwei Chen, Huachun Pan, Shumin Yang, Wenke Cheng

**Affiliations:** 1 Department of Cardiovascular Medicine, Nanchang People’s Hospital (The Third Hospital of Nanchang), Jiangxi, China; 2 College of Veterinary Medicine, Huazhong Agricultural University, Wuhan, China; 3 State Key Laboratory of Agriculture Microbiology, College of Veterinary Medicine, Huazhong Agricultural University, Wuhan, China; 4 Medical Faculty, University of Leipzig, Liebigstr 27, Leipzig, 04103 Germany

**Keywords:** Apolipoprotein B, National Health and Nutrition Examination Survey, All-cause mortality, Cardiovascular disease, Mortality

## Abstract

**Background:**

Dyslipidemia frequently coexists with hypertension in the population. Apolipoprotein B (ApoB) is increasingly considered a more potent predictor of cardiovascular disease (CVD). Abnormal levels of serum ApoB can potentially impact the mortality risk.

**Methods:**

The prospective cohort study employed data from the National Health and Nutrition Examination Survey (NHANES), which was performed between 2005 and 2016, with follow-ups extended until December 2019. Serum ApoB concentrations were quantified using nephelometry. In line with the NHANES descriptions and recommendations, the reference ranges for ApoB concentrations are 55–140 and 55–125 mg/dL for men and women, respectively. Participants were categorized into low, normal, and high ApoB levels. The low and high groups were combined into the abnormal group. In this study, all-cause mortality (ACM) and CVD mortality (CVM) were the endpoints. Survey-weighted cox hazards models were used for evaluating the correlation between serum ApoB levels and ACM and CVM. A generalized additive model (GAM) was employed to examine the dose-dependent relationship between ApoB levels and mortality risk.

**Results:**

After a median of 95 (interquartile range: 62–135) months of follow-up, 986 all-cause and 286 CVD deaths were recorded. The abnormal ApoB group exhibited a trend toward an elevated risk of ACM in relative to the normal group (HR 1.22, 95% CI: 0.96–1.53). The risk of CVM was elevated by 76% in the ApoB abnormal group (HR 1.76, 95% CI: 1.28–2.42). According to the GAM, there existed a nonlinear association between serum ApoB levels and ACM (*P* = 0.005) and CVM (*P* = 0.009).

**Conclusions:**

In the US hypertensive population, serum Apo B levels were U-shaped and correlated with ACM and CVM risk, with the lowest risk at 100 mg/dL. Importantly, abnormal Apo B levels were related to an elevated risk of ACM and CVM. These risks were especially high at lower Apo B levels. The obtained findings emphasize the importance of maintaining appropriate Apo B levels to prevent adverse outcomes in hypertensive individuals.

**Supplementary Information:**

The online version contains supplementary material available at 10.1186/s12872-024-03949-1.

## Introduction

Globally, cardiovascular disease (CVD) is the main reason for mortality and disability, highlighting the necessity for its prevention and prompt treatment [1]. Established risk factors such as diabetes, hypertension, and dyslipidemia exert significant roles in the occurrence of cardiovascular events. These conditions often coexist, resulting in 'risk factor clustering', and significantly increasing cardiovascular risk [2]. The cumulative effect of these concurrent risk factors exceeds their individual effects. The presence of two or more mildly elevated risk factors may be comparable to or exceed the cardiovascular risk posed by a single severely elevated risk factor [3]. Therefore, it is essential to view these conditions not as isolated factors but as interconnected contributors to cardiovascular risk. This perspective can explain why, despite ongoing efforts to halt the progression of CVD and optimize treatments, the incidence and prevalence of CVD-related complications remain high [4].

As the most common cardiovascular risk factor globally, hypertension influences an estimated 1.28 billion people [5]. The INTERHEART study revealed that a single risk factor could increase cardiovascular risk by two to threefold. By contrast, individuals with concurrent hypertension, type 2 diabetes, dyslipidemia, and smoking are exposed to an increased risk that exceeds 20-fold [6]. This accumulative and compounded risk poses a formidable threat to global public health. A substantial body of epidemiological evidence indicates a significant correlation between dyslipidemia and the onset of hypertension [7–10]. For a long time, low-density lipoprotein cholesterol (LDL-C) has been considered a reliable predictor of atherosclerosis and the primary target of pharmacological intervention for dyslipidemia-related parameters [11]. Despite successful control of LDL-C levels, patients with metabolic syndrome and inflammation continue to show a significant residual cardiovascular risk [12]. As established by the American Heart Association (AHA) and the American College of Cardiology (ACC), the 2018 clinical practice guidelines on cholesterol placed emphasis on the superior predictive capability of ApoB in relative to LDL-C for CVD [13]. ApoB molecules are carried by a variety of lipoproteins involved in the development of atherosclerosis, such as chymosin, LDL cholesterol and lipoprotein (a) particles. ApoB measurements predominantly reflect the sum of all potentially atherogenic lipoproteins [14]. Abnormal levels of lipoprotein B can accelerate the process of atherosclerosis, which aggravates high blood pressure and its complications, thereby increasing the risk of death in hypertensive patients. Therefore, we hypothesized that abnormal serum lipoprotein B levels may influence the risk of death. Therefore, this study was aimed at exploring the relationship between ApoB and all-cause mortality (ACM) and CVD mortality (CVM) in hypertensive populations, thus providing novel theoretical foundations and therapeutic strategies for preventing and treating CVD.

## Methods

### Study design and subjects

The current prospective cohort utilized NHANES data from 2005 to 2016, with extended follow-up through December 2019. NHANES is a complex, multi-stage, stratified probability survey conducted every two years. The data are verified and maintained by the National Centre for Health Statistics (NCHS) of the US. In this survey, we include at-home, mobile examination center (MEC) interviews, physical exams, and laboratory testing. The sampling methodologies and data acquisition procedures have previously been described in detail [15]. NHANES is a publicly accessible data platform, and most of the data is readily accessible except restricted data. The investigation was approved by the Institutional Review Board of the NCHS. All the participants were required to obtain written informed consent.

Totally 34,963 US adults participated in the six NHANES survey cycles from 2005 to 2016. The participants included in each cycle were enrolled for the first time and had the unique coded IDs. Initial identification included 6,275 hypertensive adults with recorded ApoB parameters. Then, one participant without follow-up data, two pregnant women, and 869 cancer patients were excluded. Therefore, 5,384 adults are included in this study.

### Hypertension diagnosis

The diagnosis of hypertension was established using self-reported medical histories, medication histories obtained through a self-administered questionnaire during the clinic visit, and three distinct blood pressure measurements.

1). Hypertension diagnosed by a physician.

2). Antihypertensive medication use.

3). The average systolic blood pressure (SBP) and diastolic blood pressure (DBP) are considered to be elevated if they are equal to or greater than 140 mmHg or 90 mmHg, respectively, as determined by a minimum of three measurements.

### ApoB measurements

Each participant made an appointment for the laboratory test, at which time the venous blood samples were drawn and transported to standardized laboratories, where ApoB was measured via nephelometry. Different equipment was utilized across different cycles (2005–2006: Dade Behring BN100 nephelometer, Deerfield, IL; 2007–2014: ProSpec nephelometer, Marburg, Germany; 2015–2016: Roche/Hitachi Cobas 6000 Analyzer, IN, USA.). The implementation of the Centers for Disease Control and Prevention Lipid Standardization Program by NHANES was performed to guarantee the attainment of precise and accurate measurements across different laboratories and over varying periods. ApoB levels from 2005 to 2016 were adjusted using NHANES-recommended factors in order to explain cycle-specific variations in the laboratory and device. Measured coefficients of variation for ApoB ranged from 0.7% to 7.4% over six cycles (2005–2006: 1.5%-2.9%; 2007–2008: 2%-6.2%; 2009–2010: 2.9%-4.6%; 2011–2012: 2.4%-5.8%; 2013–2014: 0.7%-7.4%; 2015–2016: 2.1%-2.7%). To ensure accurate comparisons between old and new calibrators/reagents, each reagent and calibrator lot was validated by running five to ten samples using the old calibrator/reagent run values against the new calibration channel or new reagent lot. All results must be within 5% of the old lot analysis. If the results deviate by more than 5%, the lot will not be used.

### Definition of outcomes

The investigation concentrated on two outcomes including ACM and CVM, which were determined by linking study data to the National Mortality Index through December 2019. ACM includes deaths from all causes, whereas the CVM was coded using International Classification of Diseases, Tenth Revision codes I00-I09, I11, I13 and I20-51.

### Other variables of interest

A standardized questionnaire was used to collect information in terms of age, gender, race, educational attainment, family income, tobacco and alcohol consumption, medical history, and medication use. Participants self-reported their medical histories. The NHANES Procedures Manual for Laboratory/Medical Technologists provides a comprehensive guide for measuring biochemical parameters [16]. Physical measurements such as weight, height, and blood pressure were taken at the MEC. Several variables were categorized for data integration.

i). According to the description and recommendations provided by the NHANES, the reference ranges for apoB concentrations are 55–140 and 55–125 mg/dL for men and women, respectively. Based on their serum ApoB levels, the participants were categorized into low, normal, and high types.

ii). The racial categories include white individuals who are not of Hispanic origin, black individuals who are not of Hispanic origin, individuals of Mexican American descent, and individuals of other racial backgrounds.

iii). The target audience consists of individuals who have completed education up to the ninth grade, those who have completed education from the ninth to the eleventh grade (or its high school equivalent), and individuals who have obtained a college degree or higher.

iv). Based on their smoking habits, individuals can be categorized into three groups. Never smokers are those who have smoked less than one hundred cigarettes during their lifetime. Former smokers are those who have smoked more than one hundred cigarettes during their lifetime, but are not currently smoking. Current smokers are those who have smoked more than one hundred cigarettes during their lifetime and are currently smoking, regardless of the frequency [17].

v). Individuals can be classified into four groups in line with their alcohol consumption patterns. Never drinkers (less than twelve drinks during their lifetime), former drinkers (more than twelve drinks during their lifetime, but not in the past year), light/moderate drinkers (on average, less than one drink per day for women and less than two drinks per day for men in the past year), and current heavy drinkers (on average, more than one drink per day for women and more than two drinks per day for men in the past year) [18].

### Statistical analysis

Appropriate application of MEC weights served the purpose of adjusting for oversampling, non-coverage, and non-response, thereby enhancing the accuracy of the estimates to align with the nation’s demographic composition. The baseline population is characterized by continuous variables expressed as means with standard errors (SE), while unweighted counts and weighted proportions represent categorical variables. The survey-weighted chi-square test was used for detecting disparities in categorical variables among individuals with low, normal, and high apoB levels. Given that most continuous variables were not consistent with a normal distribution, the survey-weighted Kruskal–Wallis test was used to identify the differences in ApoB levels.

A Kaplan–Meier analysis, accompanied by the log-rank test, was performed during the observation period to examine the cumulative hazard risk in hypertensive individuals with different levels of ApoB. Cox hazards models were fitted to determine the hazard ratios (HRs) and their 95% confidence intervals (CIs) for correlations between ApoB and the risk of ACM and CVM. Baseline variables exhibiting between-group differences (P < 0.1) were potential predictors for multiple regression models. To prevent overfitting, excluding those variables with a VIF of 5 or higher, the variance inflation factor (VIF) was employed to quantify multicollinearity between variables. Then, confounding covariates were incorporated into the models. Model 1 was adjusted for race, gender, and education level. Model 2 was adjusted for model 1 plus body mass index (BMI), poverty income ratio, high-density lipoprotein cholesterol (HDL), triglycerides, SBP, and DBP. Model 3 was adjusted for model 2 plus coronary heart disease, hyperlipidemia, heart failure, diabetes mellitus, heart attack, and stroke. Model 4 was adjusted in model 3 plus glucose-lowering drugs and lipid-lowering drugs. Subgroup analyses were conducted, stratifying the data by gender, age, and BMI. A generalized additive model (GAM) was employed to visually assess the dose-dependent relation between serum ApoB and the mortality risk. To verify the stability of the findings, sensitivity analyses were conducted in three ways. First, we excluded participants with less than two years of follow-up to reduce the effect of potential reverse causality. Second, taking into account the effects of statins, GLP-1 receptor agonists, and insulin on cholesterol levels, these covariates were adjusted for as variables of lipid-lowering and glucose-lowering therapy in multivariate Cox regression models. Finally, all confounders at baseline were included in the multivariate Cox regression model. All analyses were conducted using R (version 4.2) and EmpowerStats (version 4.1). A two-tailed *P* value which was less than 0.05 was regarded to be of statistical significance.

## Results

This study contained 5,384 NHANES participants between 2005 and 2016, with a mean age of 57.8 years, representing an estimated 32,759,060 hypertensive individuals. In line with their ApoB levels, participants were divided into three groups including low, normal, and high. The respective counts for each group were 191, 4743, and 450. After a median of 95 (interquartile range: 62–135) months of follow-up, the low, normal, and high A poB groups experienced 60, 847, and 79 deaths, respectively. No significant differences existed in age, estimated glomerular filtration rate, the prevalence of alcohol and tobacco consumption, as well as the utilization of antihypertensive medications among the three groups (*P* values > 0.1). Table 1 presents the detailed baseline demographic characteristics.

**Table 1:** Differences in survey-weighted baseline characteristics of stroke patients in different serum ApoB level groups

	**Low level** **(*N* = 191)**	**Normal level** **(*N* = 4743)**	**High level** **(*N* = 450)**	***P*-value**
Representative sample size	960,662	28,868,807	2,930,140	
Age (years)	53.95 (1.99)	55.75 (0.34)	55.24 (0.68)	0.466
PIR	2.46 (0.15)	2.93 (0.05)	2.69 (0.11)	< 0.001
BMI (kg/m^2^)	30.28 (0.59)	31.22 (0.15)	31.70 (0.44)	0.064
eGFR (mL/min/1.73m^2^)	83.46 (2.54)	87.11 (0.51)	85.54 (1.26)	0.249
HDL (mmol/L)	1.50 (0.05)	1.37 (0.01)	1.30 (0.02)	0.003
TC (mmol/L)	3.29 (0.06)	4.94 (0.02)	6.99 (0.04)	< 0.001
LDL (mmol/L)	1.38 (0.03)	2.86 (0.02)	4.62 (0.05)	< 0.001
TG (mmol/L)	0.90 (0.04)	1.59 (0.03)	2.61 (0.10)	< 0.001
SBP (mmHg)	129.06 (1.41)	131.33 (0.38)	136.73 (1.61)	0.002
DBP (mmHg)	67.14 (1.42)	72.54 (0.31)	75.97 (0.87)	< 0.001
Statin use (%)	52 (23.94)	955 (21.59)	35 (8.46)	< 0.001
GLP-1 receptor agonists use (%)	3 (1.97)	29 (0.93)	2 (0.60)	0.508
Insulin use (%)	27 (16.11)	250 (4.51)	25 (4.08)	< 0.001
**Gender**				< 0.001
Women	85 (46.27)	2289 (47.90)	313 (67.43)	
Men	106 (53.73)	2454 (52.10)	137(32.57)	
**Race**				0.003
Non-Hispanic white	73 (62.92)	2009 (68.27)	194 (69.81)	
Non-Hispanic black	77 (24.32)	1237 (13.81)	104 (12.22)	
Mexican American	16 (4.88)	635 (6.25)	70 (7.10)	
Other races	25 (7.88)	862 (11.68)	82 (10.88)	
**Education Levels**				0.032
Less than 9th grade	29 (12.67)	612 (7.22)	77 (11.00)	
9-11th grade/high school grade or equivalent	81 (41.50)	1937 (37.88)	170 (38.90)	
College graduate or above	76 (45.82)	2136 (54.89)	201 (50.10)	
**Congestive heart failure**				0.030
No	161 (90.08)	4390 (95.16)	426 (95.94)	
Yes	24 (9.92)	280 (4.84)	23 (4.06)	
**Diabetes mellitus**				0.072
No	109 (60.40)	3133 (71.65)	278 (69.12)	
Yes	82 (39.60)	1610 (28.35)	172 (30.88)	
**Stroke**				< 0.001
No	158 (85.65)	4435 (94.48)	421 (94.51)	
Yes	33 (14.35)	308 (5.52)	29 (5.49)	
**Heart attack**				0.068
No	164 (87.86)	4383 (93.40)	423 (94.52)	
Yes	27 (12.14)	360 (6.60)	27 (5.48)	
**Hyperlipidemia**				< 0.001
No	67 (33.37)	935 (18.58)	1 (0.09)	
Yes	124 (66.63)	3808 (81.42)	449 (99.91)	
**Coronary heart disease**				0.004
No	160 (86.01)	4426 (93.69)	421 (93.97)	
Yes	31(13.99)	317 (6.31)	29 (6.03)	
**Alcohol user**				0.782
Never	30 (13.64)	696 (12.70)	76 (15.19)	
Former	43 (18.96)	1046 (19.90)	103 (21.17)	
Mild/moderate	64 (36.69)	1413 (37.02)	116 (32.12)	
Heavy	37 (30.71)	1211 (30.38)	117 (31.53)	
**Smoking**				0.325
Never	89 (45.26)	2391 (49.42)	241 (51.97)	
Former	58 (28.85)	1376 (30.79)	114 (25.40)	
Current	40 (25.89)	935 (19.79)	92 (22.63)	
**Antihypertensive medication**				0.385
No	54 (36.45)	1660 (36.02)	184 (40.47)	
Yes	137 (63.55)	3083 (63.98)	266 (59.53)	
**Glucose-lowering drugs**				0.010
No	135 (71.61)	3792 (83.34)	364 (85.27)	
Yes	56 (28.39)	951 (16.66)	86 (14.73)	
**Lipid-lowering drugs**				< 0.001
No	93 (51.16)	3102 (66.00)	354 (80.68)	
Yes	98 (48.84)	1641 (34.00)	96 (19.32)	

Low levels < 55mg/dL, normal levels (55–125 mg/dL for women and 55–140 mg/dL for men), high levels (> 125mg/dL for women and > 140 mg/dL for men)

Continuous variables are expressed as weighted mean (Standard error, SE)

Categorical variables are expressed as counts (weighted %)

*NHANES* National Health and Nutrition Examination Survey, *PIR* Poverty income ratio, *BMI* Body mass index, *HB* Hemoglobin, *TC* Total cholesterol, *LDL* Low-density lipoprotein cholesterol, *TG* triglycerides, *HDL* High-density lipoprotein cholesterol, *SBP* Systolic blood pressure, *DBP* Diastolic blood pressure, *eGFR* Estimated glomerular filtration rate

### Associations between ApoB and all-cause mortality

**Fig. 1:**
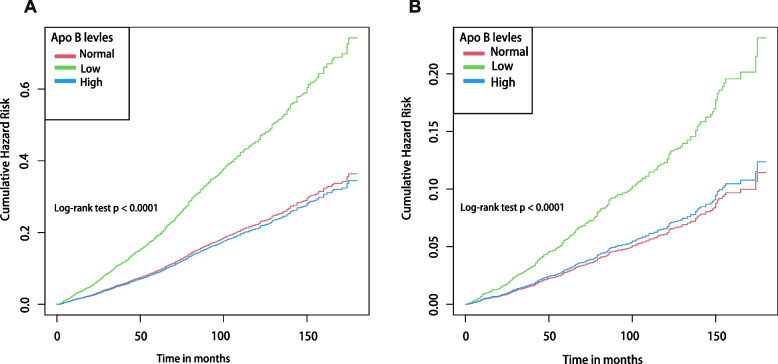
**A** Kaplan–Meier survival curve for all-cause mortality risk in different serum ApoB level groups. **B** Kaplan–Meier survival curve for the risk of CVD mortality in the different serum ApoB level groups. In the multivariate model the HRs have been fully adjusted for gender, race, education levels, poverty income ratio, body mass index, total cholesterol, high-density lipoprotein cholesterol, systolic blood pressure, diastolic blood pressure, coronary heart disease, hyperlipidemia, congestive heart failure, diabetes mellitus, stroke, heart attack, glucose-lowering and lipid-lowering drugs

As shown in Fig. 1A, the Kaplan–Meier curves revealed a statistically significant difference in ACM risk among the low, normal, and high ApoB groups over the observation period (*P* for log-rank test 0.0001), with the low ApoB group exhibiting a higher risk of ACM than the other two groups. The low ApoB group exhibited a 1.27-fold higher risk of ACM than the normal group (HR 2.27, 95% CI: 1.63–3.31; *P* < 0.001; Table 2) in the unadjusted Cox regression model. The aforementioned trend remained consistent across Models 1 to 3, even after accounting for various independent variables through progressive adjustment. Following full adjustments in Model 4, the low ApoB group maintained a 66% elevated risk of ACM in relative to the normal group (HR 1.66, 95% CI: 1.23 to 2.23; *P* < 0.001; Table 2). Nevertheless, in relative to the normal group, the elevated ApoB group did not exhibit a substantially elevated risk of ACM (all *P* values > 0.05).

**Table 2:** Weighted univariate and multivariate Cox regression to assess the association between different serum ApoB levels and the risk of all-cause and CVD mortality in stroke patients

	**Normal ApoB level**	**Low ApoB level**	**p-value**	**High ApoB level**	***p*-value**
**All-cause Mortality**					
Number of deaths	847	60		79	
Unadjusted	1	2.27 (1.65–3.11)	< 0.001	1.01 (0.77–1.30)	0.961
Model 1	1	2.23 (1.62–3.07)	< 0.001	0.94 (0.73–1.23)	0.665
Model 2	1	1.93 (1.38–2.69)	< 0.001	0.91 (0.66–1.24)	0.542
Model 3	1	1.72 (1.28–2.31)	< 0.001	0.95 (0.68–1.34)	0.774
Model 4	1	1.66 (1.23–2.24)	< 0.001	1.0 (0.71–1.40)	0.983
**CVD Mortality**					
Number of deaths	243	17		26	
Unadjusted	1	3.03 (1.99–4.62)	< 0.001	1.16 (0.74–1.81)	0.520
Model 1	1	2.89 (1.87–4.46)	< 0.001	1.09 (0.69–1.72)	0.712
Model 2	1	2.57 (1.56–4.25)	< 0.001	1.15 (0.69–1.94)	0.587
Model 3	1	2.44 (1.52–3.91)	< 0.001	1.29 (0.78–2.15)	0.319
Model 4	1	2.26 (1.40–3.63)	< 0.001	1.44 (0.87–2.40)	0.160

Model 1 adjust gender, race and education levels

Model 2 adjust Model 1 plus PIR, BMI, HDL, TG, SBP and DBP

Model 3 adjust Model 2 plus coronary heart disease, hyperlipidemia, congestive heart failure, diabetes mellitus, stroke, and heart attack

Model 4 adjust Model 3 plus glucose-lowering drugs and lipid-lowering drugs

In the subsequent analysis, the low and high ApoB groups were classified as the abnormal ApoB group. In the unadjusted model and Model 1, ACM was 29% (HR 1.29, 95% CI: 1.05–1.59; *P* = 0.015; Table 3) and 23% (HR 1.23, 95% CI: 1.05–1.59; *P* = 0.045; Table 3) higher in the abnormal group in relative to the normal group, respectively. In Models 2 and 3, the difference in risk of ACM between the abnormal and normal groups was not of statistical significance(*P* > 0.1). A trend toward an increased mortality risk from all causes was observed in the abnormal group in Model 4 (HR 1.22, 95% CI: 0.96–1.53; *P* = 0.096; Table 3). In Model 4, an upward trend in ACM risk was identified among individuals in the abnormal group (HR 1.22, 95% CI: 0.96–1.53; *P* = 0.096; Table 3).

**Table 3:** Weighted univariate and multivariate Cox regression to assess the association between different serum ApoB levels and the risk of all-cause and CVD mortality in stroke patients

	**Normal Apo B level**	**Abnormal Apo B level**	***p*-value**
**All-cause Mortality**			
Number of deaths	847	60	
Crude		1.29 (1.05–1.59)	0.015
Model 1	1	1.23 (1.0–1.50)	0.045
Model 2	1	1.19 (0.96–1.48)	0.117
Model 3	1	1.19 (0.95–1.50)	0.138
Model 4	1	1.22 (0.96–1.53)	0.096
**CVD Mortality**			
Number of deaths	243	17	
Crude		1.58 (1.17–2.14)	0.003
Model 1	1	1.50 (1.11–2.02)	0.008
Model 2	1	1.59 (1.15–2.20)	0.005
Model 3	1	1.69 (1.23–2.32)	0.001
Model 4	1	1.76 (1.28–2.42)	< 0.001

The abnormal ApoB group contains both low and high ApoB groups

Model 1 adjust gender, race and education levels

Model 2 adjust Model 1 plus PIR, BMI, HDL, TG, SBP and DBP

Model 3 adjust Model 2 plus coronary heart disease, hyperlipidemia, congestive heart failure, diabetes mellitus, stroke, and heart attack

Model 4 adjust Model 3 plus glucose-lowering drugs and lipid-lowering drugs

**Fig. 2:**
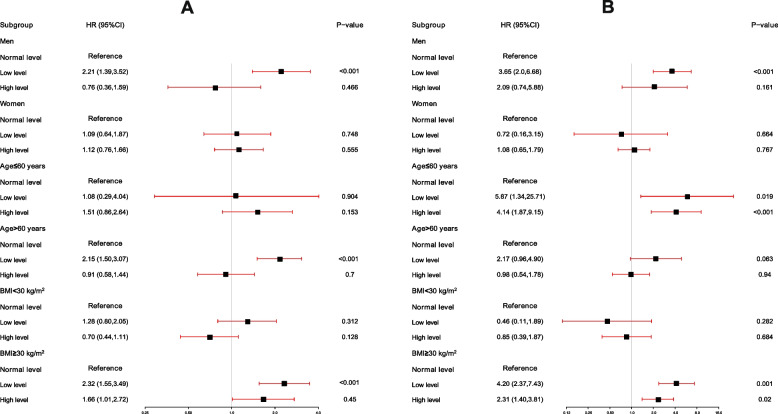
**A** Subgroup analysis of the association between different serum ApoB levels and the risk of all-cause mortality (hazard ratios, 95% CIs). **B** Subgroup analysis of the association between different serum ApoB levels and the risk of CVD mortality (hazard ratios, 95% CIs). The normal group was used as the reference group. The HRs have been fully adjusted for gender, race, education levels, poverty income ratio, body mass index, total cholesterol, high-density lipoprotein cholesterol, systolic blood pressure, diastolic blood pressure, coronary heart disease, hyperlipidemia, congestive heart failure, diabetes mellitus, stroke, heart attack, glucose-lowering and lipid-lowering drugs

Subgroup analyses were performed based on sex, age, and BMI level, as presented in Fig. 2A. In the subgroups of men, age > 60 years, and BMI ≥ 30 kg/m^2^, participants with low levels of ApoB had a 115% to 132% elevated risk of ACM. As shown in the other subgroups, the risk of ACM was comparable to that of the normal group in the low and high ApoB groups (*P* > 0.05).

### Associations between ApoB and CVD mortality

The Kaplan-Meyer curves for CVM were similar to those for ACM, showing a significant risk difference among the low-, normal-, and high-ApoB groups (*P* for log-rank test 0.0001; Fig. 1B). Participants in the low ApoB group revealed a higher risk of CVM when compared with the other two groups. As displayed in the unadjusted Cox regression model, the risk of CVM in the low ApoB group was 2.03-fold higher than that in the normal group (HR 3.03, 95% CI: 1.99–4.08; *P* < 0.001; Table 2). Even after full adjustment in model 4, the risk of CVM in the low ApoB group was still 126% higher than in the normal group (HR 2.26, 95% CI: 1.40–3.40; *P* < 0.001; Table 2). However, in all models, the high ApoB group exhibited no correlation with the elevated risk of CVM in relative to the normal group (all *P* values > 0.05).

The unadjusted model showed a 58% higher risk of CVM in the ApoB abnormal group than that in the normal group (HR 1.58, 95% CI: 1.17–2.14; *P* = 0.003; Table 3). The result was consistent across Models 1 to 4, where the risk of CVM in the abnormal group was increased by 50% (HR 1.50, 95% CI: 1.11–2.02), 59% (HR 1.59, 95% CI: 1.15–2.20), 69% (HR 1.69, 95% CI: 1.23–2.32), and 76% (HR 1.76. 95% CI: 1.28–2.42).

In subgroup analyses, an elevated risk of CVM was observed in participants with lower ApoB levels in the subgroup of men, age ≤ 60 years, and BMI ≥ 30 kg/m^2^. As depicted in Fig. 2B, the increased risk ranged from 3.65 to 4.87-fold. Compared to the normal group in the other subgroups, CVM risk between the low and high groups presented no statistically significant difference (*P* > 0.05).

### Dose-dependent relationship

**Fig. 3:**
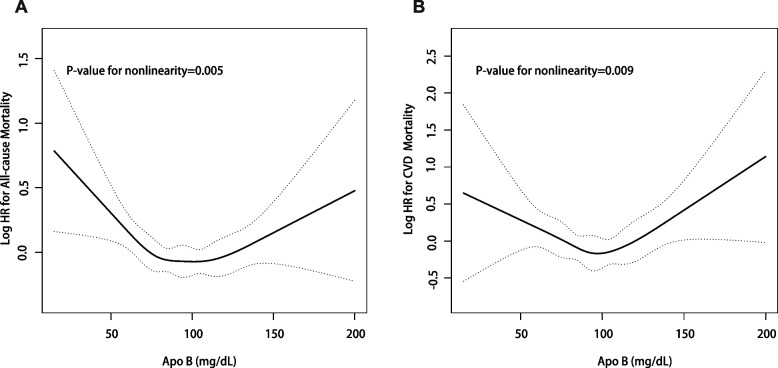
**A** Smoothing spline of the relationship between serum ApoB levels and risk of all-cause mortality. **B** Smoothing spline of the relationship between serum ApoB levels and risk of CVD mortality. Smoothing splines were performed by a generalized additive model, and adjusted for the following covariates: gender, race, education levels, poverty income ratio, body mass index, total cholesterol, high-density lipoprotein cholesterol, systolic blood pressure, diastolic blood pressure, coronary heart disease, hyperlipidemia, congestive heart failure, diabetes mellitus, stroke, heart attack, glucose-lowering and lipid-lowering drugs

As depicted in Fig. 3, the relationship between ApoB levels and ACM (P for nonlinearity = 0.005) and CVM (P for nonlinearity = 0.009) in the hypertensive population was U-shaped. ACM and CVM risk was the lowest with ApoB levels being around 100 mg/dL. In instances where ApoB levels were below 100 mg/dL, there was a consistent decline in risk with the increase of ApoB levels. By contrast, when ApoB levels exceeded 100 mg/dL, there was an observed elevation in the mortality risk as ApoB levels increased.

### Sensitivity analyses

Sensitivity analyses were performed by three strategies, and the results consistently showed a higher risk of all-cause and CVD mortality in the lower ApoB group compared with the normal group (Table S1). Similarly, abnormal ApoB levels were associated with a significantly increased risk of CVD mortality compared to the normal group (Table S2). These results are consistent with the main study.

## Discussion

This is the first study to explore the relationship between serum ApoB levels and the risk of ACM and CVM in individuals with hypertension. Our findings suggest that abnormal ApoB levels are independently related to a higher risk of ACM and CVM, especially in individuals with lower ApoB levels. There was a U-shaped relationship between serum ApoB levels and the risk of ACM and CVM, with the lowest risk around 100 mg/dL.

Dyslipidemia contributes substantially to atherosclerosis, and LDL-C is a widely studied indicator. As the protein component of plasma lipoproteins, ApoB-containing apolipoproteins play a causal role in the onset of atherosclerosis [19]. Non-HDL cholesterol refers to the cumulative quantity of cholesterol in lipoprotein particles containing ApoB [20]. Overemphasis on LDL-C at the expense of other apolipoprotein cholesterol may underestimate the atherosclerosis risk in specific populations [21]. The recent findings from the Framingham study did not identify LDL-C as a crucial risk factor for coronary artery disease [22]. A US population-based cross-sectional study found no significant association between LDL-C and the progression of atherosclerotic disease, suggesting that LDL-C might not be the optimal target for lipid-lowering therapies [23]. The INTERHEART study compared the disparities in cardiovascular risk between ApoB and non-HDL-C, suggesting that ApoB was a superior predictor of cardiovascular events [6]. Both the 2021 guidelines for CVD by the European Society of Cardiology [24] and the 2022 atherosclerotic CVD risk assessment practice statement by the American Society for Preventive Cardiology (ASPC) [25] underscored the superiority of ApoB over LDL-C in assessing cardiovascular events, due to ApoB’s effectiveness as a representative for the total concentration of dense lipoprotein particles.

In addition to the dyslipidemia observed in the normal population, monogenic dyslipidemias (MDs) are a spectrum of inherited conditions resulting from genetic anomalies in lipoprotein metabolism, leading to atypical levels of plasma lipids and lipoproteins, including LDL-C, HDL-C, and triglycerides [26]. One of the most frequent outcomes of MDs is the early onset of atherosclerosis, closely linked to elevated concentrations of LDL-C [27]. Familial hypercholesterolemia (FH), an autosomal dominant genetic disorder, emerges as the most prevalent and often underdiagnosed condition within MDs [28]. In populations where there is a high clinical suspicion of FH (indicated by a Simon Broome DFH or Dutch Lipid Clinic Network score of > 8), the prevalence of a monogenic cause ranges between 40 to 80%, whereas, in less suspicious cases, detection rates hover around 20%-30% [29]. Characterized by lifelong elevated LDL-C levels, FH manifests in two primary clinical forms: heterozygous FH (HeFH) and homozygous FH (HoFH), both attributable to mutations in the LDL receptor (LDLR), APOB, and PCSK9 genes [30]. In HeFH, pathogenic mutations in any one of these genes can instigate the disorder. It is reported that over 90% of HeFH cases involve loss-of-function mutations in the LDL receptor gene, 5%-10% are due to specific mutations in the LDLR binding domain of the APOB gene, and less than 1% result from gain-of-function mutations in the PCSK9 gene [31]. The cornerstone of treatment for FH, particularly for hypercholesterolemia, involves statin therapy. Recognized globally as the most frequently prescribed medications, statins are deemed both safe and effective [32]. Sensitivity analyses conducted in this study, incorporating statin use into the Cox regression model, yielded consistent results, underscoring the independent association between abnormal ApoB levels and the higher risk of all-cause and CVD mortality, irrespective of statin usage.

Arterial stiffness is a major contributor to human aging, and abnormal ApoB levels increase atherosclerosis and cardiac load, potentially increasing the risk of CVD and death. Nevertheless, understanding the relationship between ApoB and mortality needs to be improved. Stettler C. et al. demonstrated that when ApoB levels exceeded 0.96 g/L in patients suffering from type 1 diabetes, the risk of ACM doubled, and the risk of CVM skyrocketed by as much as sevenfold [33]. In 2021, Johannesen C discovered that ApoB concentrations predicted ACM more accurately than LDL-C in a cohort of 13,015 high cardiovascular risk individuals [34]. In a study performed in 2022, Li H et al. discovered a positive linear relationship between elevated ApoB concentrations and an increased long-term ACM risk among a cohort of patients diagnosed with coronary artery disease [35]. Notably, they also discovered that low ApoB levels at baseline were related to a high risk of a poor prognosis, possibly due to malnutrition. ApoB is the leading protein component of LDL, occupying approximately 95% of the total protein component of LDL. Johannesen et al. reported a U-shaped relationship between LDL-C levels and ACM risk in the general individuals, with the lowest risk at a level of 140 mg/dL [36].

Cheng Q et al. analyzed the relationship between non-HDL cholesterol and ACM and CVM in hypertensive populations from 1999 to 2014 in the NHANES dataset [37]. They discovered a U-shaped relationship between non-HDL cholesterol and mortality, consistent with the obtained findings. ApoB is a product of non-HDL cholesterol. According to this study, lower ApoB levels are correlated with an elevated risk of ACM and CVM. Full adjustment for multiple variables did not affect these results. In addition, most models showed that abnormal ApoB levels exhibited an association with an elevated risk of ACM and CVM. The observation was confirmed by the dose–response analysis, revealing a U-shaped relationship between ApoB levels and the risk of ACM and CVM. With ApoB levels being approximately 100 mg/dL, ACM and CVM risk was the lowest. Given that current guidelines do not specify an optimal range for ApoB, the obtained results suggest that either too high or too low ApoB levels can negatively affect survival outcomes.

In subgroup analyses, abnormal ApoB levels are strongly associated with mortality risk in men and obese populations. The exact mechanism behind this is still unknown. However, several preliminary explanations can be made. At first, women may be less at risk for cholesterol-related mortality because estrogen could lower cholesterol and help prevent vascular disease in premenopausal women [38]. Second, obese individuals often have multiple coexisting chronic diseases, including diabetes and cardiovascular disease. These patients often receive early pharmacological interventions, including using GLP-1 receptor agonists and statins, which can effectively improve lipid levels. This could lower the effect of lipid levels on mortality risk.

### Clinical implications and further perspectives

The study uncovers that abnormal ApoB levels, particularly low levels, showed a correlation with the elevated ACM and CVM in hypertensive individuals. This association suggests that maintaining optimal ApoB levels may be critical in reducing mortality risk. The current work also reveals a non-linear correlation between ApoB levels and mortality risk, with the lowest risk at an ApoB level of around 100 mg/dL. Men and obese populations appear to be more impacted by abnormal ApoB levels. Therefore, healthcare providers may need to pay particular attention to maintaining appropriate ApoB levels in these subgroups. These insights emphasize the potential value of incorporating ApoB monitoring into personalized, risk-based strategies to manage hypertensive patients.

Furthermore, these results suggest that health strategies tailored to an individual’s specific ApoB level could be beneficial. For instance, if ApoB levels are too high or too low, interventions such as lifestyle modifications or specific medications could bring ApoB levels to an optimal range. These personalized strategies, aimed at maintaining appropriate ApoB levels, may improve patient outcomes by lowering the associated mortality risks. While this study points to the potential effectiveness of such tailored interventions, further research is required to verify these findings and establish a cause-and-effect relationship.

### Limitations

However, this study still has the following limitation. First, the inability to establish a causal relationship between ApoB and mortality risk was attributed to the observational nature of this investigation. Second, it should be noted that the cohort utilized was exclusively derived from the US, which may limit the generalization of the results of this study to other diverse populations. The United States is a multiethnic country, which contributes to the heterogeneity of the cohort populations and influences the results. Third, the exposure factor in this study was circulating levels of ApoB. In the human circulatory system, the predominantly detected form of ApoB is ApoB-100. Due to limitations in the raw data, subclasses of ApoB could not be further analyzed. In addition, apolipoprotein A-related analyses could not be performed because apolipoprotein A measurements were not included in the NHANES laboratory testing protocol. Finally, multiple models with iterative adjustments for distinct variables were performed in the regression models to confirm the stability of the results. The effect of residual variables remains unknown. In addition, various generations and operators may have influenced the results of ApoB assays. Although NHANES corrected the results, this effect could not be ruled out.

## Conclusions

In the US hypertensive population, serum ApoB levels were U-shaped and related to ACM and CVM risk, with the lowest risk at 100 mg/dL. Importantly, abnormal ApoB levels showed a relationship to the elevated risk of ACM and CVM, and the risks were especially high at lower ApoB levels. These findings emphasize the importance of maintaining appropriate ApoB levels to prevent adverse outcomes in these individuals.

### Supplementary Information

Supplementary Material 1.

## Data Availability

Data are publicly available at https://www.cdc.gov/nchs/nhanes/index.htm.
