# Supplementary material for: Relationship between serum apolipoprotein B and risk of all-cause and cardiovascular disease mortality in individuals with hypertension: a prospective cohort study
Source: BMC Cardiovasc Disord. 2024 May 24;24:273. doi: 10.1186/s12872-024-03949-1 (PMC11127391; doi:10.1186/s12872-024-03949-1)
Supplement: Supplementary file 1 — Supplementary Material 1. [file 12872_2024_3949_MOESM1_ESM.pdf]

# Supplement

**Table S1. Sensitivity analyses of the risk of all-cause and CVD mortality in the low and high ApoB groups compared with the normal group.**

| Exclusion of participants within 2 years [a] | Low ApoB level | | High ApoB level | |
|---|---|---|---|---|
| **All-cause Mortality** | 1.84 (1.30-2.61) | < 0.001 | 0.84 (0.58-1.21) | 0.350 |
| **CVD Mortality** | 2.67 (1.59-4.47) | < 0.001 | 1.39 (0.83-2.35) | 0.211 |
| **Adjusted for statins, GLP-1 receptor agonists, and insulin [b]** | | | | |
| **All-cause Mortality** | 1.59 (1.16-2.18) | 0.004 | 1.0 (0.71-1.39) | 0.988 |
| **CVD Mortality** | 2.30 (1.38-3.85) | 0.001 | 1.42 (0.83-2.41) | 0.196 |
| **Adjusted for all confounders at baseline [c]** | | | | |
| **All-cause Mortality** | 1.50 (1.11-2.02) | 0.008 | 1.06 (0.75-1.51) | 0.729 |
| **CVD Mortality** | 2.14 (1.34-3.42) | 0.002 | 1.46 (0.87-2.43) | 0.152 |

[a] indicates that the multivariate Cox model was adjusted for gender, race, education levels, PIR, BMI, HDL, TG, SBP, DBP, coronary heart disease, hyperlipidemia, congestive heart failure, diabetes mellitus, stroke, heart attack, glucose-lowering drugs, and lipid-lowering drugs.

[b] indicates that the multivariate Cox model was adjusted for gender, race, education levels, PIR, BMI, HDL, TG, SBP, DBP, coronary heart disease, hyperlipidemia, congestive heart failure, diabetes mellitus, stroke, heart attack, statins, GLP-1 receptor agonists, and insulin.

[c] indicates that the multivariate Cox model was adjusted for gender, race, education levels, eGFR, PIR, BMI, HDL, TG, SBP, DBP, coronary heart disease, hyperlipidemia, congestive heart failure, diabetes mellitus, stroke, heart attack, glucose-lowering drugs, lipid-lowering drugs, statins, antihypertensive medication, GLP-1 receptor agonists, insulin, alcohol user and smoking.

| Table S2. Sensitivity analyses of the risk of all-cause and CVD mortality in the ApoB abnormal compared with the normal group. | | |
|---|---|---|
| **Exclusion of participants within 2 years [a]** | **Low ApoB level** | |
| **All-cause Mortality** | 1.16 (0.89-1.51) | 0.283 |
| **CVD Mortality** | 1.87 (1.31-2.66) | < 0.001 |
| **Adjusted for statins, GLP-1 receptor agonists, and insulin [b]** | | |
| **All-cause Mortality** | 1.19 (0.94-1.51) | 0.140 |
| **CVD Mortality** | 1.75 (1.26-2.44) | < 0.001 |
| **Adjusted for all confounders at baseline [c]** | | |
| **All-cause Mortality** | 1.21 (0.94-1.56) | 0.134 |
| **CVD Mortality** | 1.73 (1.26-2.38) | < 0.001 |

[a] indicates that the multivariate Cox model was adjusted for gender, race, education levels, PIR, BMI, HDL, TG, SBP, DBP, coronary heart disease, hyperlipidemia, congestive heart failure, diabetes mellitus, stroke, heart attack, glucose-lowering drugs, and lipid-lowering drugs.

[b] indicates that the multivariate Cox model was adjusted for gender, race, education levels, PIR, BMI, HDL, TG, SBP, DBP, coronary heart disease, hyperlipidemia, congestive heart failure, diabetes mellitus, stroke, heart attack, statins, GLP-1 receptor agonists, and insulin.

[c] indicates that the multivariate Cox model was adjusted for gender, race, education levels, eGFR, PIR, BMI, HDL, TG, SBP, DBP, coronary heart disease, hyperlipidemia, congestive heart failure, diabetes mellitus, stroke, heart attack, glucose-lowering drugs, lipid-lowering drugs, statins, antihypertensive medication, GLP-1 receptor agonists, insulin, alcohol user and smoking.
